# A Mechanistic Physiologically-Based Biopharmaceutics Modeling (PBBM) Approach to Assess the In Vivo Performance of an Orally Administered Drug Product: From IVIVC to IVIVP

**DOI:** 10.3390/pharmaceutics12010074

**Published:** 2020-01-17

**Authors:** Marival Bermejo, Bart Hens, Joseph Dickens, Deanna Mudie, Paulo Paixão, Yasuhiro Tsume, Kerby Shedden, Gordon L. Amidon

**Affiliations:** 1 Department of Pharmaceutical Sciences, College of Pharmacy, University of Michigan, 428 Church Street, Ann Arbor, MI 48109-1065, USA; mbermejo@goumh.umh.es (M.B.); bart.hens@kuleuven.be (B.H.); deanna.mudie@lonza.com (D.M.); ppaixao@ff.ulisboa.pt (P.P.); yasuhiro.tsume@merck.com (Y.T.); 2 Department of Engineering, Pharmacy Section, Miguel Hernandez University, San Juan de Alicante, 03550 Alicante, Spain; 3 Department of Pharmaceutical & Pharmacological Sciences, KU Leuven, Herestraat 49, 3000 Leuven, Belgium; 4 Department of Statistics, University of Michigan, Ann Arbor, MI 48109, USA; josephdi@umich.edu (J.D.); kshedden@umich.edu (K.S.); 5 Global Research and Development, Lonza, Bend, OR 97703, USA; 6 Research Institute for Medicines (iMed.ULisboa), Faculty of Pharmacy, Universidade de Lisboa, Avenida Professor Gama Pinto, 1649-003 Lisboa, Portugal; 7 Merck & Co., Inc., 126 E Lincoln Ave, Rahway, NJ 07065, USA

**Keywords:** oral absorption, in silico modeling, GastroPlus, Phoenix WinNonlin, pharmacokinetics, clinical studies, ibuprofen, manometry, gastrointestinal, mechanistic modeling, PBPK, PBBM

## Abstract

The application of in silico modeling to predict the in vivo outcome of an oral drug product is gaining a lot of interest. Fully relying on these models as a surrogate tool requires continuous optimization and validation. To do so, intraluminal and systemic data are desirable to judge the predicted outcomes. The aim of this study was to predict the systemic concentrations of ibuprofen after oral administration of an 800 mg immediate-release (IR) tablet to healthy subjects in fasted-state conditions. A mechanistic oral absorption model coupled with a two-compartmental pharmacokinetic (PK) model was built in Phoenix WinNonlinWinNonlin^®^ software and in the GastroPlus™ simulator. It should be noted that all simulations were performed in an ideal framework as we were in possession of a plethora of in vivo data (e.g., motility, pH, luminal and systemic concentrations) in order to evaluate and optimize these models. All this work refers to the fact that important, yet crucial, gastrointestinal (GI) variables should be integrated into biopredictive dissolution testing (low buffer capacity media, considering phosphate versus bicarbonate buffer, hydrodynamics) to account for a valuable input for physiologically-based pharmacokinetic (PBPK) platform programs. While simulations can be performed and mechanistic insights can be gained from such simulations from current software, we need to move from correlations to predictions (IVIVC → IVIVP) and, moreover, we need to further determine the dynamics of the GI variables controlling the dosage form transit, disintegration, dissolution, absorption and metabolism along the human GI tract. Establishing the link between biopredictive in vitro dissolution testing and mechanistic oral absorption modeling (i.e., physiologically-based biopharmaceutics modeling (PBBM)) creates an opportunity to potentially request biowaivers in the near future for orally administered drug products, regardless of its classification according to the Biopharmaceutics Classification System (BCS).

## 1. Introduction

Although advances have been made and insights have improved throughout the years, there is still a lot of gastrointestinal (GI) variables that are poorly understood that should be investigated for their influence on drug release and systemic exposure after oral intake of a drug product [1,2]. The knowledge has improved about the intestinal behavior of an active pharmaceutical ingredient (API) in terms of solubility, dissolution, permeation, supersaturation, and precipitation, as demonstrated in different clinical aspiration studies performed over the last ten years [3,4,5,6,7]. In these studies, drug concentrations were measured in healthy volunteers after aspiration of GI fluids after oral administration. Subsequently, drug concentrations were determined in these aspirates in parallel with collecting blood samples to assess systemic exposure. As these studies contributed to formulation behavior in the GI tract, it was not always straightforward to correlate the measured drug concentrations in the upper part of the small intestine with concentrations appearing in blood. The knowledge about the impact of the surrounding dynamic GI environment on drug- and formulation behavior remains rather scarce and requires further investigation. The constantly changing climate of GI pH and motility patterns can alter drug behavior along the GI tract in such a way that it is necessary to investigate these mechanisms and, in a next step, to take these variables into account in in vitro and in silico predictive models to facilitate oral drug development [8,9,10,11]. GI motility is defined by the different contractile phases of the migrating motor complex (MMC): phase I is an inert period with little activity; phase II features sporadic contractions gradually ascending in magnitude; and phase III is characterized by powerful, high-frequency contractile bursts that promote emptying of contents where peak flow rates are observed [12]. In recent work, a clinical aspiration study was performed that aimed to measure the impact of physiological variables on the systemic exposure of orally-administered ibuprofen (immediate-release tablets, 800 mg) [13,14]. The outcome of this study demonstrated how phase III contractions and fluctuating pH (caused by the low buffer capacity) in the human intestinal tract had a major impact on ibuprofen’s dissolution and, consequently, absorption in fasted (*n* = 20) and fed state (*n* = 17).

Based on these new insights, it has become clear that working in a biorelevant setting (i.e., simulated GI media, multi-compartmental in vitro models, solubility/permeability interplay) will result in more accurate predictions. From that perspective, the OrBiTo community took the initiative to design a decision tree which makes it handy for formulation scientists to select the most appropriate biopredictive dissolution test depending on the biopharmaceutical properties of the drug compound and the type of formulation [15]. This decision tree clearly focuses on some biorelevant aspects of the GI tract that play a pivotal role in and have a significant impact on the luminal behavior of a drug product; these variables should not be neglected in a biopredictive dissolution test. For instance, in the case of weakly basic compounds, the implementation of a GI transfer should be included in order to capture the supersaturated state of the drug after transfer from the stomach compartment to the intestinal compartment. Besides the optimization of in vitro tools, mechanism-based in silico models should be optimized and validated at the same time. The outcome of the in vitro dissolution tests can serve, in a second step, as input for physiologically-based pharmacokinetic (PBPK) platforms to simulate the systemic exposure of the drug. While a lot of progress has been made by mechanism-based in silico models to identify key issues in the development of new oral drug products [11,16,17,18,19], there are still many aspects that are poorly understood that need to be optimized/integrated to maximize the utility of these models towards predicting the systemic outcome of novel and generic drug candidates. Commercially available software packages such as the Simcyp^®^ simulator, GastroPlus^™^, and PK-Sim^®^ are just a few programs that are frequently used in the non-clinical stage of drug product development to get an idea about the in vivo performance of the drug product when administered to patients. The underlying syntax/algorithm of these packages describes the mass transport of the drug throughout the different built-in compartments and should be adequately reflecting the physiological processes of the human body. From an academic perspective, it is our mission to see (i) if the underlying mathematical equations are making any sense and (ii) if they are representing the physiological variables in a proper and biorelevant manner (physiological range). For instance, all these programs describe the stomach compartment as a single, well-stirred compartment, assuming that a drug will be homogeneously distributed along the entire stomach after oral administration. Based on measured gastric concentrations of the non-absorbable markers, phenol red and paromomycin, it was clearly shown that these markers were not homogeneously distributed among the different regions of the stomach (i.e., fundus, body and antrum). Therefore, we developed a mechanistic oral absorption model in the Berkeley-Madonna^®^ software package (Version 8.3.18) that could only explain the observed luminal data when the stomach was handled as a two-compartmental model that was connected with a bypass flow to reflect the immediate fast transfer of liquid from stomach to small intestine after drinking a solution of these markers [20].

For this study, we aimed to reflect the luminal and systemic concentrations of ibuprofen under fasting state conditions starting with the simplest model, assuming a first-order kinetic process for dissolution, gastric emptying and absorption. In a second step, the model was revised, and dissolution was handled as pH dependent and gastric emptying was treated as a first-order process until the time of appearance of phase 3 contractions post-dose—after which, the remaining dose was directly transferred to the duodenal compartment. The mechanistic model focused on the integration of phase III contractions to simulate a house-keeper wave that is responsible for the direct release of ibuprofen particles from the stomach into the small intestine. In the different compartments of the small intestine, the dissolution of ibuprofen is driven by the regional pH, determining the fraction dissolved and undissolved. Afterward, a statistical analysis was performed to see how both scenarios matched with the observed luminal and systemic concentrations. In addition to this model, an advanced compartmental absorption and transit (ACAT^™^) model was developed in GastroPlus^™^ to assess the impact of dynamic pH, fluid volumes and gastric emptying on the systemic performance of ibuprofen. A comparison of these simulations was made with simulations performed by default settings.

## 2. Materials and Methods

### 2.1. Reference Intraluminal and Systemic Data of Ibuprofen

#### 2.1.1. Intraluminal and Systemic Profiling of Ibuprofen in Healthy Volunteers

The study was held at the University of Michigan Hospital after receiving approval by the internal review board (IRB) at both University of Michigan and FDA (HUM00085066) under the project (HHSF223201310144C (Sun D. and Amidon G.L., Principle Investigators)—09/30/15–12/31/18 “Modernization of in vivo-in vitro Oral Bioperformance Prediction and Assessment: A research study to evaluate the performance of an ibuprofen oral dosage form in the gastrointestinal tract of healthy adult volunteers”) [13,14]. Briefly, 13 healthy volunteers (men and women) were recruited; 7 out of 13 subjects participated in the study twice to generate intra-subject variability data. All volunteers provided written informed consent to participate in this study. After a fasting period, a multi-lumen GI tube from MUI Scientific (Mississauga, ON, Canada) was introduced via the mouth to the small intestine. Abdominal fluoroscopy was performed to ensure the GI tube was properly positioned in the different regions of the GI tract (i.e., stomach, duodenum, proximal and distal jejunum). The subject was asked to remain in bed while the GI tube was equilibrated by performing a baseline GI motility test for approximately 3–5 h (Medical Measurement Systems (MMS), Williston, VT, USA). Prior to the administration of the ibuprofen tablet, an intravenous catheter was introduced in the antecubital area of the subject for blood collection. The catheter was kept open with a heparin and saline solution. The subjects were asked to empty his/her bladder prior to the start of the study. At approximately 4:00 AM, the subject was given a single oral dose of ibuprofen (800 mg tablet). The study drug was administered with 250 mL of water containing USP grade phenol red (0.1 mg/mL). The actual amount of water consumed was measured and recorded. Volunteers were not obliged to drink the total amount of administered water to avoid any feeling of nausea at the start of the study. GI samples (stomach, duodenum and jejunum) were collected at 0, 0.25, 0.5, 0.75, 1, 1.5, 2, 2.5, 3, 4, 5, 6, and 7 h. Blood samples (4 mL/time point) were collected at 0, 0.167, 0.33, 0.5, 0.75, 1, 1.5, 2, 2.5, 3, 4, 5, 6, 7, 8, 12 and 28 h. Plasma was separated from blood samples by centrifugation and stored at −80 °C until analysis. The pH of GI fluid samples was immediately measured and recorded. The GI fluid samples were centrifuged at a speed of 17,000× *g* for 10 min and the supernatant was placed in the new tube for drug concentration analysis.

#### 2.1.2. Recording of Post-Dose Phase III Contractions

MMC phase III motility periods were identified from the water-perfused manometric measurements using spectral density estimation and penalized logistic regression as described in detail by Hens and co-workers and will be briefly discussed here [13]. After positioning, the catheter was connected to a computer console that generated real-time manometry recordings in the different segments of the GI tract (Medical Measurement Systems, Dover, NH, USA). The manometric channels attached to the catheter were perfused with water at a rate of 2 mL/min and served as intestinal pressure recording ports to assess intestinal motility. Each segment contained four motility channels to monitor pressure events. Baseline intestinal motility was evaluated for 3–5 h prior to study drug administration of the tablet. Subsequently, GI motility was measured continuously for 7 h. Powerful antral phase III contractions were defined as the occurrence of regular 2–3 contractions per minute for at least 2 min with an average amplitude of 75 mmHg. Duodenal phase III contractions were characterized by 11–12 contractions per minute with an average amplitude of 33 mmHg which can last for at least 3 min. As the contractile activity propagates, it becomes less spatiotemporally organized resulting in slower propulsion rates in the distal small bowel. The corresponding spectral density estimate of a phase III period will have high energy levels in the 10–12 cycles/min components, leading to a concentrated spectrum. During non-phase III motility, the spectral density will have a more diffuse spectrum. Using penalized logistic regression, it was clearly observed that the proportion of energy in the 9–12 cycle/min frequencies is an important predictor of phase III motility.

#### 2.1.3. Thermodynamic Equilibrium Solubility of Ibuprofen in Fasted-State Human Gastric and Intestinal Fluids (Fahgf/Fahif)

The thermodynamic solubility of ibuprofen was determined by the shake-flask method (25 RPM), incubating gastrointestinal fluids for 24 h with an excess amount of ibuprofen (Acros Organics, Morris Plains, NJ, USA) at 37 °C. The fluids that were used for measuring the thermodynamic solubility of ibuprofen were aspirated gastric, duodenal and jejunal fluids of three different time points of subject B005-F2. Following the 24 h incubation, samples were centrifuged for 15 min at 17,000 *g* (AccuSpin Micro 17, Fisher Scientific, Pittsburgh, PA, USA). The supernatant was diluted 10-fold with methanol (Fisher Scientific, Pittsburgh, PA, USA) and again centrifuged for 5 min in order to discard any proteins that could interfere with the HPLC analysis (see below). Solubility measurements were performed in triplicate.

#### 2.1.4. Bioanalysis of Ibuprofen by HPLC

Solubility samples were analyzed by HPLC–UV (Hewlett Packard series 1100 HPLC Pump, Santa Clara, CA, USA), combined with Agilent Technologies 1200 Series Autosampler (Santa Clara, CA, USA). A volume of 5 µL was injected into the HPLC system connected to a UV lamp that was able to detect ibuprofen at a wavelength of 220 nm (Agilent 1100 Series UV lamp, Santa Clara, CA, USA). An isocratic run containing 70% acetonitrile (VWR International, West Chester, PA, USA) and 30% purified water (both containing 0.1% TFA) was used to detect ibuprofen at a retention time of 2.9 min using a reversed-phase C-18 column (Eclipse Plus C18, 4.6 × 150 mm, 5.5 µm, Agilent Technologies) and a 1 mL/min flow rate. The calibration curve was made in methanol based on a stock solution of ibuprofen in methanol (1 mM). Linearity was observed between 10.32 µg/mL and 0.32 µg/mL. The observed peaks were integrated using ChemStation software (Agilent Technologies, B.04.03 version). The developed analytical method met the FDA requirements for bioanalytical method validation [21].

### 2.2. Mechanistic Oral Absorption Modeling in Phoenix WinNonlin^®^

#### WinNonlin User-Customized Mechanistic Model to Stress the Pivotal Underlying GI Variables: InVivo_GIS versus InVivo_GISPlus

A compartmental model including stomach, duodenum and jejunum with first-order transit and absorption rates was designed to describe the time evolution of ibuprofen mass and concentrations in duodenum, jejunum and plasma. The following assumptions were made with respect to the ‘in vivo Gastrointestinal System model (InVivo_GIS)’:

1. Ibuprofen dissolution was considered negligible in the stomach chamber due to the acidic pH (pH < pKa). The administered oral tablet disintegrates in the stomach and particles will not be dissolved but emptied in the next segment, i.e., the duodenum. In addition, no significant absorption can occur from the stomach.
2. Gastric emptying follows a first-order kinetic process.
3. Dissolution follows a first-order process in the duodenal and jejunal segment. The dissolution rates are proportional to the remaining amount of solid ibuprofen.
4. Duodenal and jejunal compartments are well-mixed, resulting in homogenous drug concentrations.
5. The permeability of the intestinal membrane is high for ibuprofen, indicating that dissolved ibuprofen will be immediately absorbed. Only solid particles transit from the duodenum to the jejunum.
6. Transit from the duodenum to the jejunum is faster than the transit from the jejunum to the more distal parts.
7. Drug degradation does not occur in the GI lumen.

This basic model was further extended to explore the influence of intestinal pH and motility. Therefore, we developed a model which we will refer to as the ‘InVivo_GISPlus’:

1. Gastric emptying follows a first-order kinetic up to the next post-dose phase III contractions when all the remaining stomach content is suddenly emptied in the duodenum.
2. Dissolution follows a first-order process in the duodenal and jejunal segment. The dissolution rates are proportional to the remaining amount of solid ibuprofen. The dissolution rate is modeled as a function of luminal pH values. Ibuprofen solubility is re-calculated at each time point with the duodenum or jejunal pH at that specific moment.

**Figure 1:**
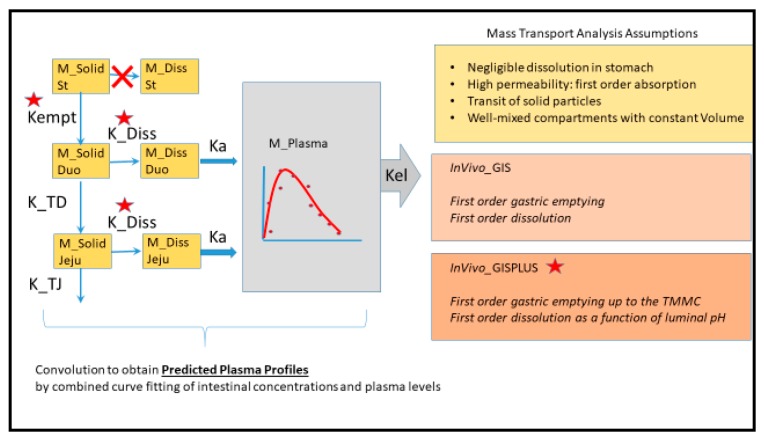
Mass transport analysis scheme and assumptions of InVivo_GIS and InVivo_GISPLUS models. *K*_empt_: first-order emptying rate coefficient; *K*_a_: first-order absorption rate coefficient; K_TD and K_TJ: first order transit coefficients from duodenum to jejunum and from jejunum to distal segments, respectively; TMMC: time to the next Phase III wave post-dose; K_Diss: first-order dissolution rate constants.

Model schemes are represented in Figure 1. The system of differential equations was written as the American Standard Code for Information Interchange (ASCII) code and run in Phoenix WinNonlin^®^ V8 (Certara, Princeton, NJ, USA).

Six differential equations were used, describing the amount of solid ibuprofen as a function of time in the stomach ($M_{stomach}^{solid}$), in the duodenum ($M_{duodenum}^{solid}$), and in the jejunum ($M_{jejunum}^{solid}$), as well as the amount of dissolved ibuprofen as a function of time in the duodenum ($M_{duodenum}^{dissolved}$), in the jejunum ($M_{jejunum}^{dissolved}$) and in plasma ($M_{plasma}^{}$). Integrated model parameters were:

- Duodenal and jejunum average fluid volume values during the sampling time, V1 and V2, respectively;
- Transit rate coefficients from the duodenal to the jejunal segment, K_TD; and from the jejunum to the more distal segment, K_TJ;
- First-order rate coefficient of gastric emptying, *K*_empt_;
- Dissolution rate coefficient, K_Diss;
- First-order absorption rate constants, *K*_a_.

In each subject, the elimination rate coefficient (*K*_el_) and the distribution volume in plasma (V3) were fixed to the value obtained after performing a non-compartmental PK analysis. Time to the next phase III wave post-dose (TMMC) was fixed to the experimentally determined value [22]. The individual pH values in duodenum and jejunum at each time point were used in InVivo_GISPlus model to recalculate ibuprofen’s solubility at each time point. The absorption rate coefficient was fixed to a high value of 12 h^−1^ based knowing that ibuprofen does not have any permeability-related issues (fraction absorbed ~1) [22]. This value was based on ibuprofen permeability in rat small intestine [23] that was scaled up to human *P*_eff_ value with the human-rat correlation described by Zakeri-Milani et al. [24]. The 13 mathematical equations to describe the mass transport of ibuprofen in the InVivo_GISPlus model are summarized in the Supplemental Information. Both models InVivo_GIS and InVivo_GISplus were fitted simultaneously to duodenal, jejunal and plasma concentrations.

### 2.3. Mechanistic Oral Absorption Modeling in GastroPlus™

#### 2.3.1. GastroPlus™ Advanced Compartmental Absorption Transit (ACAT™) Mechanistic Absorption Model

Simulations were performed by the commercially available PBPK modeling platform GastroPlus™ 9.6 (Simulations Plus, Inc., Lancaster, CA, USA) and all simulations were judged based on and compared with the observed luminal and systemic concentrations of ibuprofen after oral administration of 800 mg ibuprofen (Shreveport, LA, USA; IBU™—Ibuprofen Tablets, USP, 800 mg) to twenty healthy subjects in fasted state.

The advanced compartmental and absorption transit (ACAT^™^) was applied with slight adjustments related to pH, gastric emptying and fluid dynamics. This model is described in detail by Hens and Bolger [25]. To implement a dynamic pH and fluid volume as a function of time, a mixed-multiple dosage form was selected. The mixed-multiple dosage form consisted of 13 different .cat files, personalized by a different pH and volume value to simulate a dynamic fluid and pH model over time. The implemented pH values were the same average values according to the values as measured during the clinical aspiration study. The implemented values for the fluid volume were extracted from Mudie and co-workers [26]. The gastric transit time was set at 2.04 h, which conforms with the average time to phase III contractions post-dose, as observed in the clinical aspiration study of ibuprofen.

MedChem Designer 5.0 (Simulations Plus, Inc., Lancaster, CA, USA) was applied to draw the molecular structure of ibuprofen. Data describing the drug’s physicochemical and biopharmaceutical properties were obtained from literature or from estimates calculated by ADMET predictor 9.0 (Simulations Plus, Inc., Lancaster, CA, USA). All physicochemical and biopharmaceutics parameters that were used to perform the simulations are described in Table 1.

A two-compartmental PK model was used to describe the distribution and clearance of ibuprofen. Values for rate constants (K_10_, K_12_, K_21_) were optimized based on literature data that reported systemic data of ibuprofen after intravenous (IV) administration of 800 mg ibuprofen with an infusion rate of approximately 6 min (between 5 and 7 min) [31]. Estimations of these rate constants were performed by the PKPlus™ module.

#### 2.3.2. Data Presentation

The observed intraluminal and systemic concentration–time profiles are presented as the mean ± standard deviation (SD) for all participating subjects and are extracted from previous work [13,14]. Pharmacokinetic and intraluminal parameters are reported and compared with the simulated outcomes.

## 3. Results and Discussion

### 3.1. Mechanistic Oral Absorption Modeling in WinNonlin

**Figure 2:**
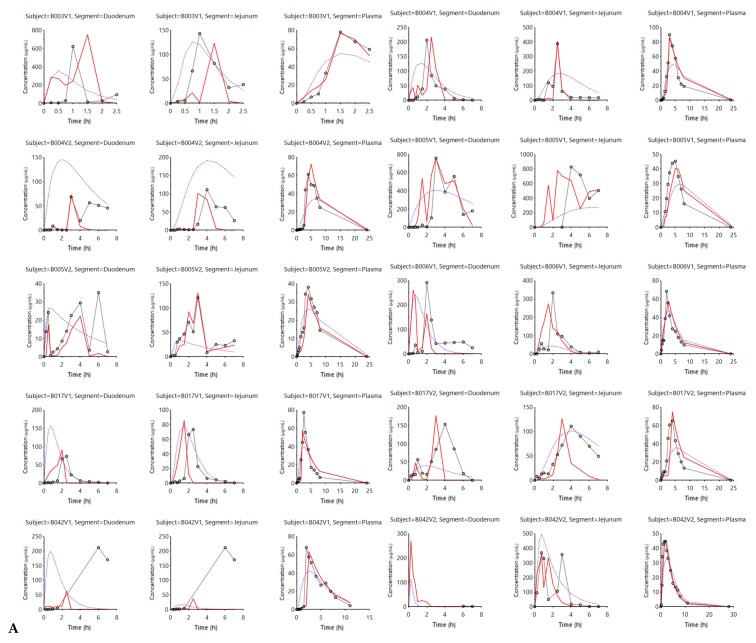

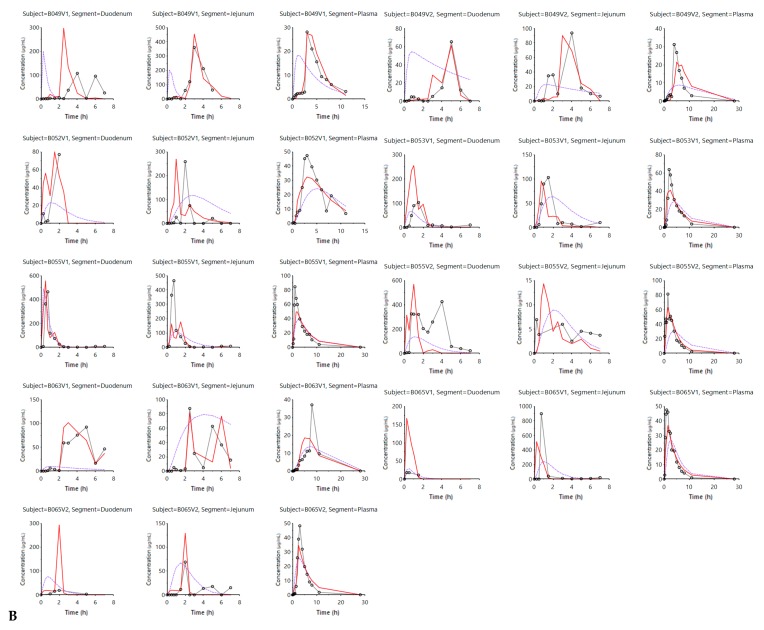
(**A**) and (**B**) describe the experimental and model-fitted concentration values of ibuprofen for all subjects. Experimental (black dots) and model-fitted concentration values of ibuprofen in the duodenal, jejunal and systemic compartments for each and every subject in fasting state conditions. Each row includes the observed and simulated concentration–time profiles in the duodenal, jejunal and plasma segments in three separate columns. The purple line reflects the InVivo_GIS model-predicted values, whereas the red line corresponds with the simulated (model-fitted) values derived from the InVivo_GISPlus model. The experimental values are reflected by the dark grey lines and dots.

Figure 2 shows the simulated outcomes (model-fitted values) when applying the InVivo_GIS (purple lines) versus the InVivo_GISPlus (red lines).

**Figure 3:**
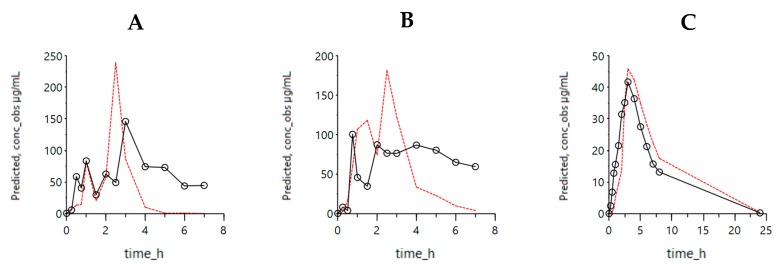
Simulated outcomes of the average concentrations using the InVivo_GISPlus model. Average experimental ibuprofen concentrations are shown by the black dots in the duodenum (**A**), jejunum (**B**) and plasma (**C**) across all the subjects. Simulated values of the InVivo_GISPlus model are represented with red lines. The experimental values are reflected by the dark black lines and dots.

When comparing both simulated profiles derived from the two models, it is clear that including luminal pH values and the ‘house-keeper’ phase III wave contractions provide better predicted (i.e., model-fitted) values. This is not only the case for the simulated plasma concentrations—capturing plasma *C*_max_—but also for the improved predictions with respect to luminal levels reflecting the oscillations associated with pH changes. Applying dynamic pH values and, therefore, showing an improved reflection of the intraluminal behavior was also observed when using the InVivo_GISPlus model to simulate the average concentration–time profiles in the duodenum, jejunum and plasma concentrations (Figure 3).

To quantitatively assess the improved predictions with the InVivo_GISPlus model, a comparison between the outcomes derived from the InVivo_GIS and InVivo_GISPlus was made and the simulated results were compared with the observed data and the prediction error was expressed as an absolute percentage deviation. Table 2 summarizes the mean absolute percentage deviation between predicted and experimental values in the main pharmacokinetic parameters (*C*_max_, *T*_max,_ and *AUC*) in the three compartments, namely the duodenum, jejunum, and plasma.

Table 3 shows the applied settings of each model parameter to adequately simulate the corresponding intraluminal and systemic concentration–time profiles for each individual.

The estimated volumes are higher than the reported values in duodenum and jejunum measured by magnetic resonance imaging (MRI) [26]. Nevertheless, it should be noted that the applied average value could be interpreted as the volume of fluid directly in contact with the solid particles during that specific period. Considering that continuous secretion and absorption of water in the small intestine occurs, the volume of fluid flowing through the segment can be high. A potential interpretation to translate the obtained parameters to the in vitro GIS device could be that the average fluid volume in contact with solid ibuprofen in duodenum and jejunum is in total 220 mL over an 8 h period to complete absorption. That would correspond to a 0.5 mL/min volumetric flow to be implemented in the in vitro GIS system.

**Figure 4:**
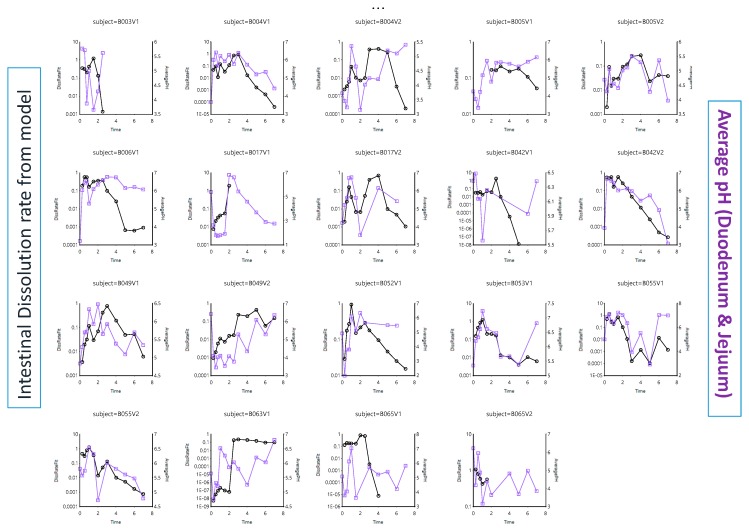
Average intestinal pH values in each subject (purple line and squares) and the intestinal dissolution rate (black line and dots) estimated from InVivo_GISPLUS model.

As ibuprofen will be heavily dependent on the pH along the GI tract to dissolve, the dissolution rate was plotted against the residual pH values. Figure 4 depicts the intestinal dissolution rate (estimated from the InVivo_GISPlus model) versus the measured average pH (duodenum and jejunum) for each individual as a function of time.

**Figure 5:**
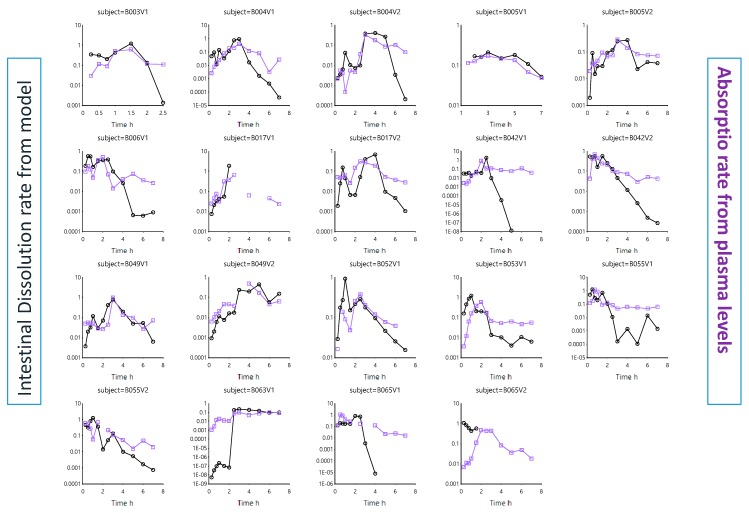
The intestinal in vivo ibuprofen dissolution rate estimated from InVivo_GISPLUS model and the absorption rate obtained from Wagner–Nelson deconvolution profiles in each individual. Absorption rates were derived from previous work by Bermejo and co-workers [22].

The in vivo dissolution rates in each subject can be estimated from the differential equations derived from the InVivo_GISPlus model. As Figure 4 shows, the pH fluctuations sometimes dictate directly the dissolution rate. The overlap is not perfect as other variables (such as the fluid volume) also affect the dissolution rate. However, in this model, a static volume was considered. If the dissolution rate increases, the amount of ibuprofen entering the systemic circulation will increase as well. Therefore, the intestinal dissolution rate was plotted against the absorption rate, deconvoluted from the plasma concentration–time profiles. This was done for each individual (Figure 5).

**Figure 6:**
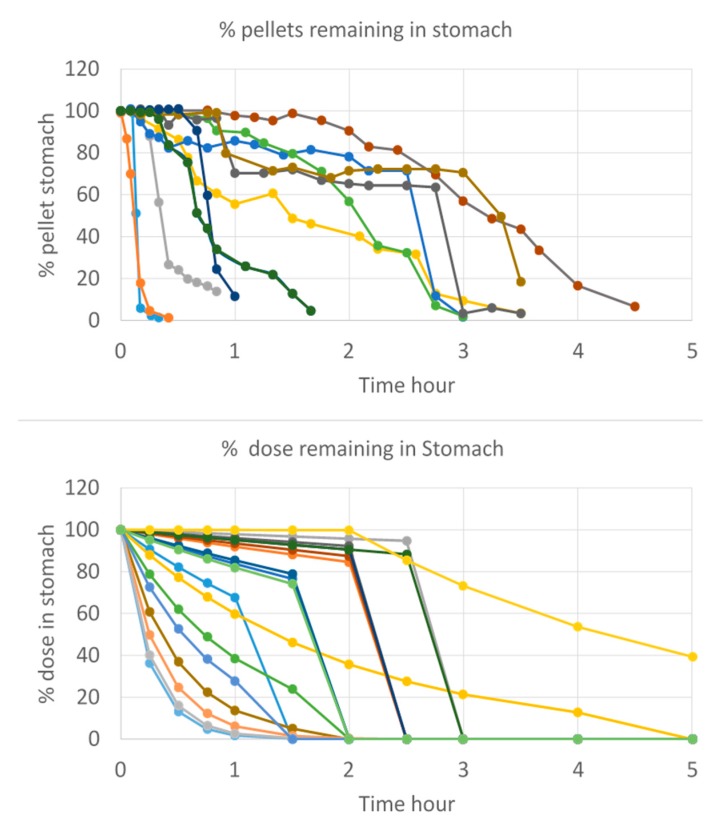
(Upper plot) Data from 19 individual scintigraphy studies of gastric emptying of pellets collected by Locatelli et al. [32]. (Bottom plot) Individual solid ibuprofen particle gastric emptying kinetics predicted by InVivo_GISPLUS model.

This work also aimed to demonstrate the impact of gastric emptying on the systemic exposure of the drug. In this case, and as shown by Hens et al., the time of phase III contractions post-dose will determine the arrival of ibuprofen in the intestinal tract. The faster these contractions will be initiated, the higher the plasma *C*_max_ will be. It is hypothesized that a fast onset of this house-keeper wave will remove more drug content directly from the stomach into the small intestine, resulting in high amount of drug that will be available for absorption (assuming pH > pKa). Whenever these phase III contractions are rather postponed (e.g., due to the intake of food), drug release from the stomach to the small intestine will be rather pulsatile than instantaneous, resulting in a lower driving force for intestinal absorption which ultimately leads to a lower plasma *C*_max_. The variability in gastric emptying of solid particles was also observed by Locatelli and colleagues when visualizing the gastric emptying process of pellets by scintigraphy studies [32]. The variability in emptying as a function of time was compared with the variability in emptying that was simulated by the InVivo_GISPlus model for each subject and demonstrated a similar trend (Figure 6).

### 3.2. Mechanistic Oral Absorption Modeling in GastroPlus™

The first commercial software program to attempt a comprehensive description of the GI tract in the context of a PBPK model was GastroPlus^™^ (Simulations Plus, Inc., Lancaster, CA, USA). The first version of GastroPlus^™^ was established in August 1998 and was based on the work of Lawrence X. Yu and Gordon L. Amidon [33]. The model consisted of “continuous stirring tank reactor” compartments to describe the transit of a drug from one segment in the GI tract to the other, with simple estimations of (i) dissolution based on aqueous solubility and (ii) absorption rate coefficients based on existing pharmacokinetic data. In 2001, an advanced compartmental absorption and transit (ACAT^™^) model was developed and defined each compartment’s volume and transit by mass balance approximations. Later on, in 2018, Hens and Bolger aimed to convert the static settings of the ACAT^™^ model to more dynamic settings [25]. They developed a dynamic fluid and pH model in the GastroPlus^™^ simulator to reflect the dynamic alternations of fluid volumes and pH values in the different compartments of the GI tract. Especially in the case of BCS class 2 compounds, suffering from poor aqueous solubility and high permeability, these dynamic settings will result in improved predictions towards the in vivo outcome of the drug product when comparing these simulations with the simulations obtained when static, default settings were applied. The benefit of these dynamic settings has already been shown for posaconazole, a weakly basic drug [25].

#### 3.2.1. Solubility versus pH: pH-Driven Dissolution

**Figure 7:**
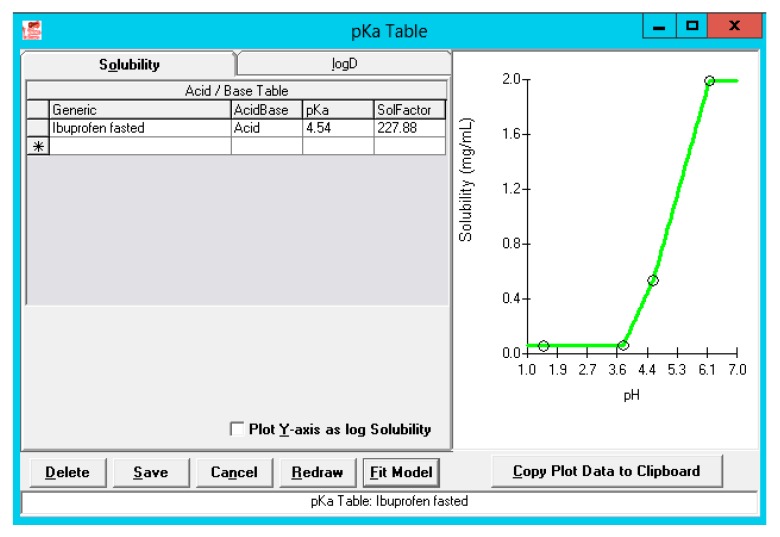
Solubility versus pH profile for ibuprofen. The green line represents the predicted solubility versus pH curve, whereas the blue dots represent the measured solubility values in fasted-state human GI fluids. The upper blue dot is a solubility value of ibuprofen measured in fasted-state duodenal fluid by Heikkilä and co-workers [28].

Thermodynamic solubility of ibuprofen was determined in three different aspirated fluids (i.e., gastric, duodenal and jejunal) of subject B005-F2. The ADMET Predictor 9.0 was used to predict the acidic pKa and how solubility would be defined in the physiological range. The observed versus simulated solubility values closely matched as depicted in Figure 7.

The solubility factor is equal to the ratio of the maximum solubility to the intrinsic solubility for this specific acidic pKa. This demonstrates the ability of ibuprofen to easily dissolve at pH levels above its pKa, converting to its ionized form which is more soluble than its non-ionized form.

It should be noted that in this clinical study, authors monitored the residual bulk pH in the GI tract (stomach, duodenum, and jejunum) and measured the solution concentrations of ibuprofen in these different regions. The pH values of the aspirates were extremely fluctuating as a consequence of the low buffer capacity [13]. As research scientists in pharmaceutical industry don’t have any access to these values and mostly make use of high buffer capacity media to explore drug dissolution, overestimations in predicting the plasma *C*_max_ will be made (see below: Advanced compartmental absorption and transit simulations: static simulations with default settings). However, there is more evidence that biopredictive dissolution setting should focus on integrating relevant aspects that have a major impact on the fraction dissolved of a drug. For instance, lowering the buffer capacity of the media and highlighting the interplay between surface and bulk pH is a critical aspect that should not be neglected. This has been observed for modified-release, but also for immediate-release formulations [34,35,36,37]. Pepin and co-workers modeled the dissolution profiles of acalabrutinib (weak base, pKas 3.54 (B), 5.77 (B), 12.1 (A)) using an in-house Excel^®^ tool and concluded that making use of the bulk apparent drug solubility will lead to an over-estimation of the drug dissolution rate at all pH values below the highest drug pKa [38]. By taking into account product particle size distribution (P-PSD) and the surface pH of drug particles, accurate simulated dissolution rates were simulated. In addition, in the case of ionizable compounds such as ibuprofen, the pH at the surface of dissolving particles (pH_0_) is a complex function between buffer- and drug-related properties. In the case of ibuprofen, in vitro results demonstrated that the surface pH is lower than the bulk pH (ranging from pH 4.8–5.8) in 5 mM bicarbonate depending upon hydrodynamics which can hamper the dissolution process as such. This was observed by Al-Gousous and co-workers, who observed differences in bulk and surface pH (and thus bulk and surface solubility) resulting in slower dissolution kinetics of ibuprofen when using low bicarbonate-buffered, dissolution media [39]. Therefore, the use of the human buffer bicarbonate will be more favorable to adequately reflect the luminal dissolution kinetics and to represent the relevant interactions between buffer species and drug molecules [39,40,41]. Biopredictive dissolution tests performed by Cristofoletti et al. demonstrated that ibuprofen dissolution in a lower buffer capacity medium (i.e., 5 mM phosphate buffer) affected bulk pH and its own dissolution kinetics [42,43]. Considering P-PSD and the self-buffering capacity of ibuprofen resulted in the best simulation with respect to plasma *C*_max_ and AUC for two Nurofen^®^ tablets of 200 mg orally administered to healthy adults. In another clinical study by Hofmann and colleagues [44], ibuprofen suspensions (varying in particle size radius) were intraduodenally administered in healthy subjects. After administration, duodenal pH was monitored in parallel with systemic exposure of ibuprofen. The administration of small particles led to a more pronounced pH drop than for large particles under the same infusion conditions. Still, absorption rates were higher for these smaller particles compared to the larger particles, but no significant differences in plasma *C*_max_ were observed, suggesting that variability in the systemic outcome of the drug is more related to the rate of gastric emptying (motility-driven) and/or intestinal transit times. Besides the bulk/surface pH, the hydrodynamics of the GI tract also have an enormous impact on the dissolution rate. Performing dissolution experiments for 200 mg of ibuprofen in 5 mM phosphate buffer at 75, 50 and 30 rotations per minute (RPM) in the USP II apparatus, demonstrated maximum cumulative fractions dissolved of 0.83, 0.84 and 0.26, respectively. Based on computational fluid dynamics (CFD), the presented shear rates in the GI tract are more in line with the shear rates that are reproduced when lower rotation speeds are applied [6].

In conclusion, some important, yet crucial, GI variables should be integrated into biopredictive dissolution testing (low buffer capacity media, phosphate versus bicarbonate buffer, hydrodynamics) to account for a valuable input for PBPK platform programs.

#### 3.2.2. Simulation of Distribution and Clearance of Ibuprofen: A Two-Compartmental Pharmacokinetic (PK) Approach

**Figure 8:**
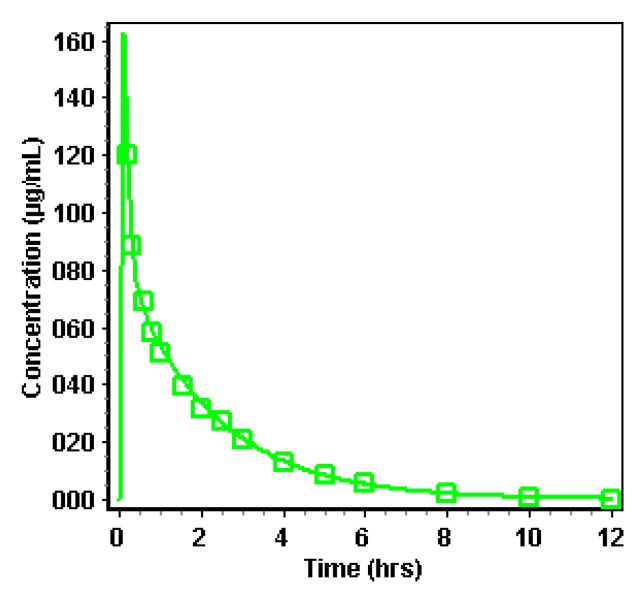
Observed (green squares) versus predicted (green line) concentrations of ibuprofen after intravenous administration of an 800 mg dose to 12 healthy subjects [31]. Simulations were performed by using a two-compartmental PK model.

Simulation of distribution and clearance of ibuprofen was performed in the PKPlus™ module based on literature data that showed the clearance of ibuprofen after an intravenous administration of an 800 mg dose of ibuprofen with a perfusion rate of approximately 6 min. For this study, 12 healthy subjects (aged between 18 and 65 years old) were recruited [31]. One-, two- and three-compartmental models were compared, and the best fit was observed for a two-compartmental PK model based on the Akaike Information Criterion (AIC) and *R*². As no extensive first-pass metabolism is observed for ibuprofen [45], a two-compartmental approach is definitely sufficient in describing the distribution and clearance of ibuprofen. Observed versus simulated data are depicted in Figure 8.

#### 3.2.3. Advanced Compartmental Absorption and Transit Simulations: Static Simulations with Default Settings

In the first set of modeling experiments, the default settings of GastroPlus™ were applied in order to assess predictions when a static volume and pH is applied in each compartment of the ACAT™ model (Table 4).

**Figure 9:**
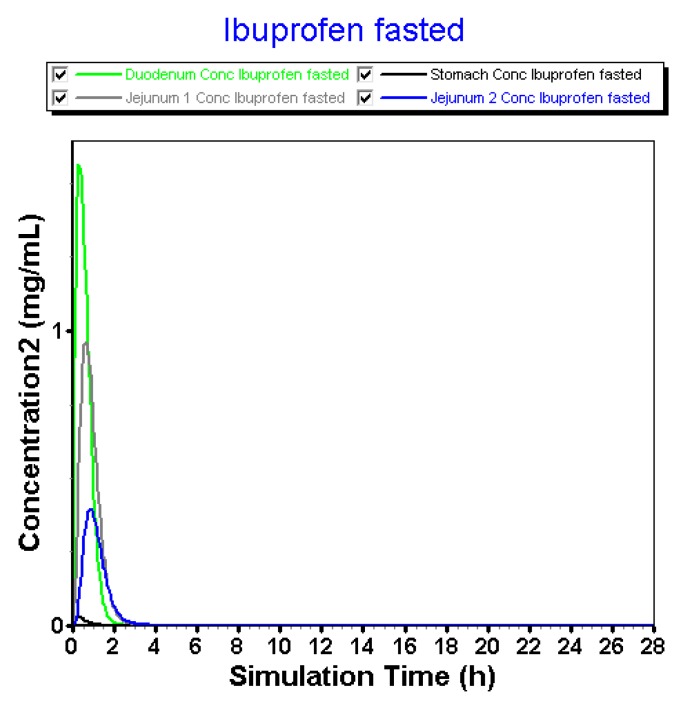
Simulated concentration–time profiles of ibuprofen in the different GI compartments of the GastroPlus™ simulator.

Regarding the fact that (i) there is no dissolution-limiting step at pH > 6 and that (ii) large volumes are always present during the simulation time, fast onset of dissolution was observed in the intestinal compartments (Figure 9).

Solution concentrations in the stomach are negligible due to the integrated acidic pH (pH 1.3) that will prevent almost any dissolution of ibuprofen in the gastric compartment. After transfer, ibuprofen will rapidly dissolve in the more neutral pH environment of the intestinal tract. There is a trend that the dissolution is the highest in the duodenum followed by the first part of the jejunum and the second part of the jejunum (diluting effect). When the 800 mg dose appears in the first compartment of the GI tract, dissolution will be enhanced due to the large volume (44.57 mL) and high pH (pH 6). As the solubility of ibuprofen is approximately 2 mg/mL at pH 6.2 (Figure 7), an enormous amount of ibuprofen will immediately dissolve and be available for absorption without any limitations. As the concentration at the surface of the ibuprofen particles (*C*_s_) drives the dissolution rate under sink conditions (pH < 6.2), whereas the bulk concentration (*C*_b_) comes into play in the case when there are no sink conditions [39].

**Figure 10:**
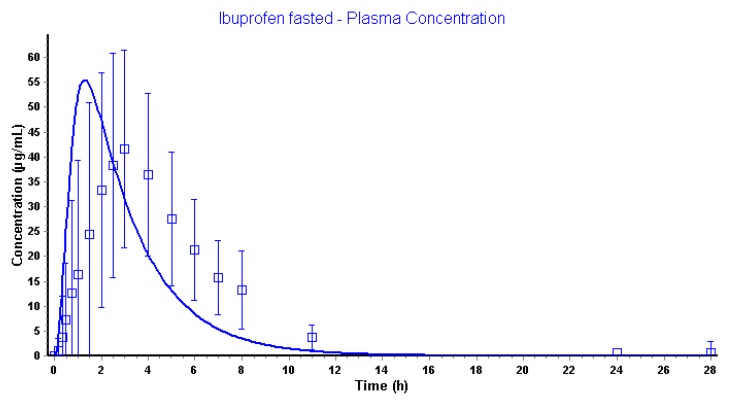
Observed (blue squares) and simulated (blue line) plasma concentrations of ibuprofen after oral administration of an 800 mg dose.

This is an ongoing process until it hits the transit time to move forward for the remaining undissolved particles towards the next compartment. Obviously, this luminal behavior will be reflected in the systemic compartment, as depicted in Figure 10.

Pharmacokinetic parameters are shown in Table 5.

**Figure 11:**
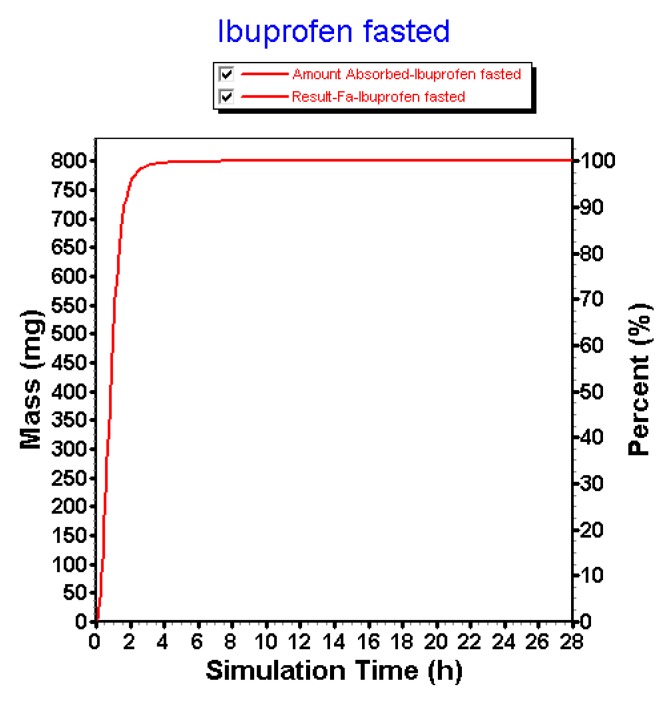
Amount absorbed of ibuprofen under the default settings in the GastroPlus™ simulator.

Using the default settings, simulations performed by GastroPlus™ are overestimating the observed pharmacokinetic parameters with respect to plasma *C*_max_ and plasma *T*_max_. Although predictions are in the same concentration range, there is still a 33% higher predicted plasma *C*_max_ compared to the observed plasma *C*_max_. Moreover, the simulated plasma *T*_max_ is appearing tremendously earlier related to the fast dissolution in the intestinal compartments. The clinical aspiration study clearly demonstrated the presence of ibuprofen in the GI tract up to 7 h. This simulation, however, informs us that the amount absorbed of ibuprofen is 100% in approximately 2 h (Figure 11).

In conclusion, there is a mismatch between the simulations and the observed data, highly related to physiological variables (i.e., pH, fluid volumes and gastric emptying) that were considered as pivotal covariates explaining intersubject variability in oral and systemic drug behavior of ibuprofen. Therefore, in the next set of simulations, these variables will be optimized.

#### 3.2.4. Advanced Compartmental Absorption and Transit Simulations: Dynamic Simulations with Adjusted Settings

In the second set of simulations experiments, the goal was to better (in terms of accuracy) reflect the intraluminal behavior taking into account some important physiological aspects that were not considered during the simulations when default settings were applied. All data derived from the clinical aspiration study were analyzed in previous work and revealed how GI motility and pH have a major impact on plasma *C*_max_ and *T*_max_, respectively. As intraluminal pH is not static at all in the human GI tract, the ACAT™ model was adjusted using a constantly changing pH as a function of time, in line with the values observed in the clinical aspiration study. This was applied to all segments of the GI tract (i.e., stomach, duodenum and jejunum). Moreover, the gastric transit time was delayed from 0.25 h to 2.04 h. As the observed house-keeper wave was on average 2.04 h [22], the gastric transit time was postponed to this specific value. The strong burst of phase III contractions is a surrogate for the rapid emptying of gastric content into the upper small intestine and was demonstrated to have an important impact on the plasma *C*_max_ of ibuprofen for this specific study.

**Figure 12:**
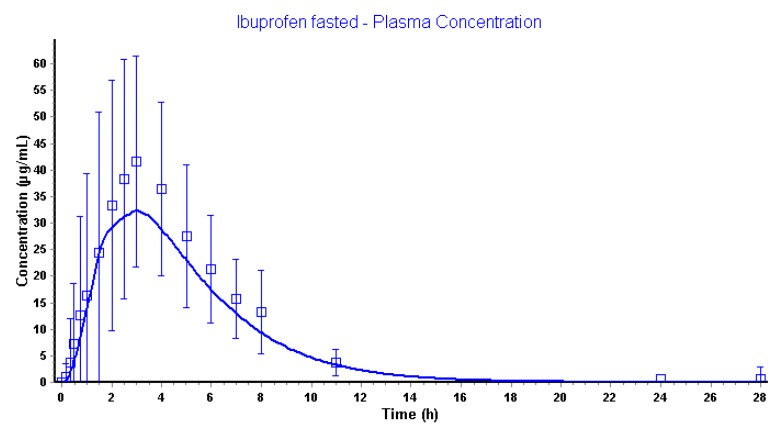
Simulated and observed plasma concentrations of ibuprofen applying the dynamic ACAT™ model.

Application of mixed-multiple doses as dosage form in GastroPlus™ makes it possible to implement different .cat files for each time point (every 15 min) all containing a different fluid volume and pH as observed by MRI data and pH values of aspirated fluids, respectively. All these separate .cat files were uploaded in the GastroPlus™ simulator and were used in chronological order. Figure 12 demonstrates the simulated and observed plasma data.

Pharmacokinetic disposition parameters are shown in Table 6.

**Figure 13:**
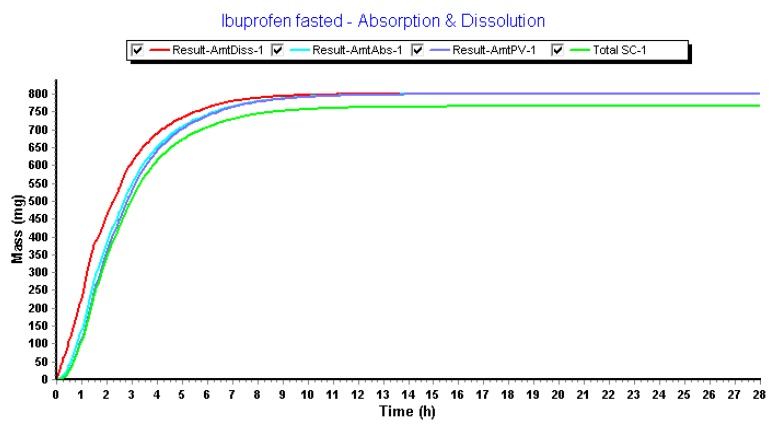
The amount dissolved, absorbed, reaching the portal vein and reaching the systemic circulation after mechanistically modeling of ibuprofen concentrations in GastroPlus™.

The application of the dynamic settings resulted in a predicted plasma *C*_max_ differing 22% compared to the observed plasma *C*_max_. The predicted plasma *T*_max_ was similar to the observed plasma *T*_max_. The exposure (expressed as the area under the curve (*AUC*)) is the same as observed for the simulations performed with the default settings, highly likely because the amount of dose that is absorbed is 100%. However, the dissolution and absorption of ibuprofen were more sustained using dynamic settings (Figure 13).

**Figure 14:**
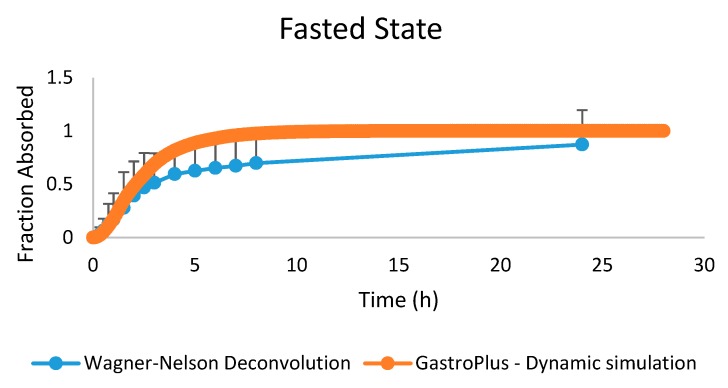
The observed fraction absorbed (based on Wagner–Nelson deconvolution) versus the simulated fraction absorbed from the GastroPlus™ simulator.

When comparing the fraction absorbed from the observed date (by deconvolution) versus the fraction observed that was simulated in GastroPlus™ using the dynamic settings, a positive overlap was observed (Figure 14).

**Figure 15:**
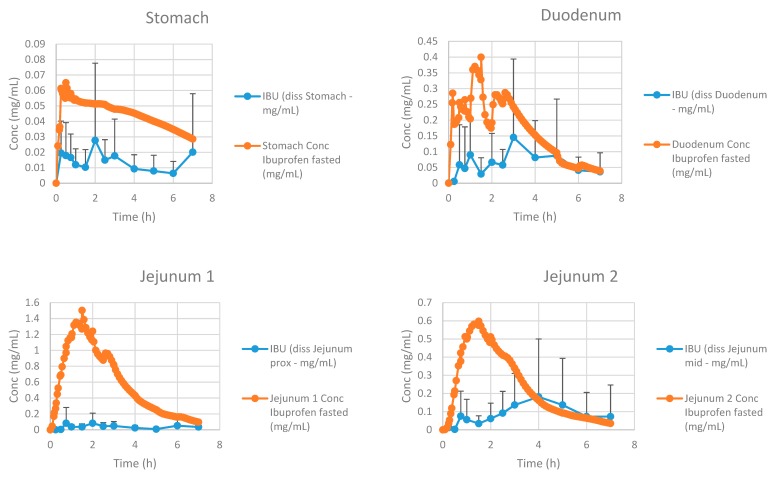
Simulated and observed intraluminal concentrations of ibuprofen in the different segments of the GI tract. Observed data are presented as the mean ± SD.

Figure 15 represents the simulated and observed intraluminal profiles in the different regions of the GI tract.

**Figure 16:**
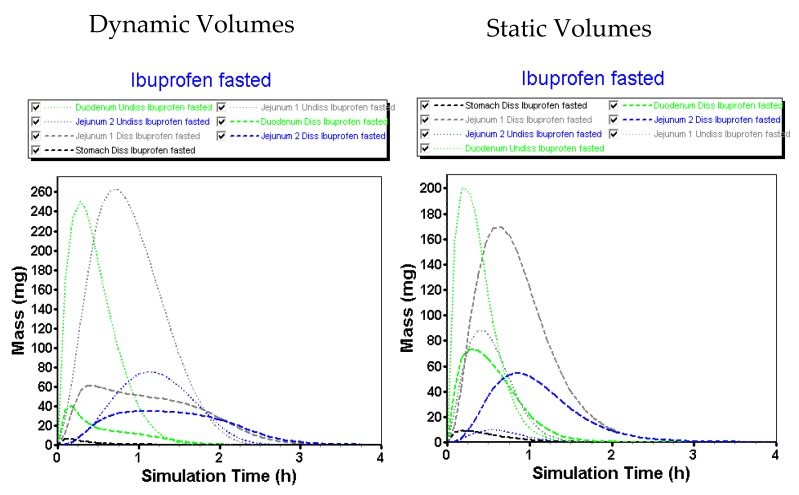
Simulated GI profiles when applying a static (i.e., constant) versus a dynamic fluid model.

Simulated results are in line with the observed data, although slightly overestimated with respect to the jejunal concentrations. The reason for this phenomenon can be attributed to the fact that GastroPlus™ handles the small intestine as different compartments, all characterized by specific transit times. As the human jejunum and ileum are quite large (2.5 m and 3.5 m, respectively), the presence of fluid pockets may play a significant role in the amount of drug that will dissolve. It is estimated that the jejunum consists of 13 mL of fluids based on the MRI data of Mudie and co-workers [26], which is more than enough for ibuprofen to dissolve. However, in vivo, these volumes are presented as small, separate water pockets that may hamper the drug to reach its equilibrium solubility. The impact of pockets is less concerned for the duodenal compartment as this segment is only 25 cm long and, based on these simulations, the absence of water pockets seems to have less impact on drug dissolution as the predictions are in line with the in vivo duodenal concentrations. Therefore, these simulations may open public debate to discuss the relevance of simulating these pockets in computational modeling as recently done by Yu and co-workers [46], who developed a dynamic fluid compartmental and absorption transit (DFCAT) model. Related to the compound characteristics (e.g., solubility and permeability), stochastic modeling of these fluid pockets could influence the predicted outcome of the drug product. Figure 16 represents the amounts of ibuprofen dissolved versus undissolved when applying a dynamic versus static fluid model (pH values of the different compartments were the same for both simulations). 

We hypothesize that the present fluids will impact the amount of drug dissolved, even though the drug has no limited capacity with respect to dissolution (BCS class 1/2a/3) and regardless of favorable pH to initiate dissolution of the drug in the intestine (BCS class 2a) [47]. Future studies should shed light on the relevance and importance of this topic.

**Figure 17:**
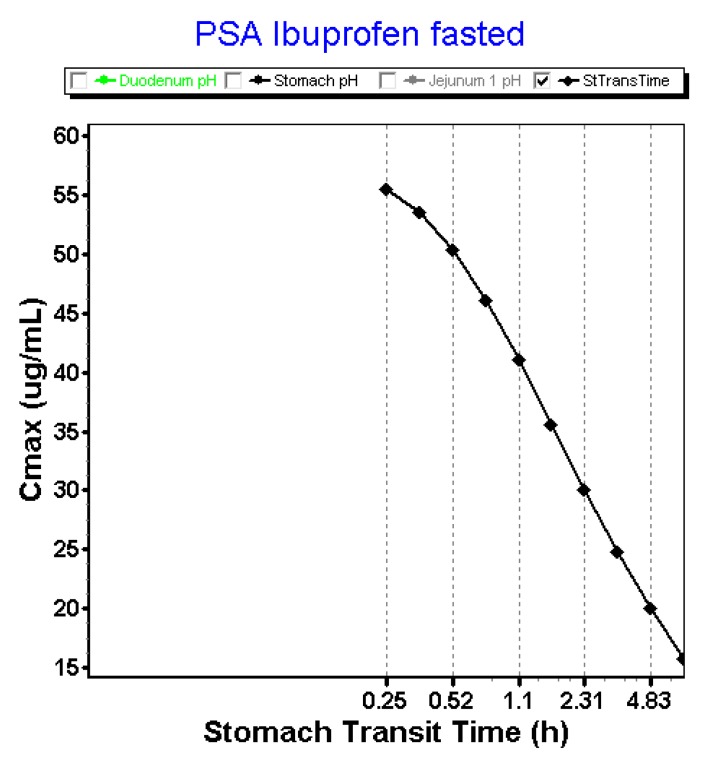
Impact of stomach transit time on the plasma *C*_max_ for ibuprofen after oral administration of an 800 mg dose in fasted state. PSA: parameter sensitivity analysis.

The rate of gastric emptying will alter systemic exposure in such a way that it is favorable to have a fast release of ibuprofen into the small intestine which will generate a high driving force for intestinal absorption. In contrast, when gastric emptying is delayed and ibuprofen will be sustained released from the stomach, the driving force will not be as big as for a direct onset of gastric emptying. Figure 17 demonstrates the parameter sensitivity analysis (PSA) with respect to the ‘gastric transit time’. These data are in line with the observed data from the clinical study where a fast onset of post-dose phase III contractions resulted in a higher plasma *C*_max_.

## 4. Conclusions and Future Directions: Requesting Biowaivers?

In conclusion, this work demonstrated a mechanistic modeling approach to explain the intraluminal and systemic performance of an orally administered ibuprofen drug product (RLD) in fasted-state conditions. The simulations that were performed highlighted the importance of considering gastric emptying, fluid volumes, motility, and pH as indispensable covariates that should be included in the models to ensure realistic predictions of systemic plasma concentrations of ibuprofen. Both these simulations were done in a user-customized model (Phoenix WinNonlin^®^) and in a commercially available software package (GastroPlus™). In both cases, we demonstrated the importance of integrating physiological variables to mechanistically understand and observe the impact of these parameters on ibuprofen intestinal absorption. With respect to fluid volumes, simulations in GastroPlus™ demonstrated the impact of dynamic fluids, as measured by an MRI study, on the dissolved amounts of ibuprofen throughout the different GI compartments. There should be further developed with respect to the ‘complexity’ of in silico models to predict the in vivo outcome plasma levels of a drug. Clear guidelines should assist formulation scientists in whether specific physiological variables should be considered and should be integrated into computational models, highly depending on the purpose of the simulation. Parameter and sensitivity analyses can serve as useful tools to assess the sensitivity of physiological variables predicting the in vivo performance of an oral drug product. Even today, the dynamics of these GI processes are relatively poorly described quantitatively and do not adequately reflect the in vivo GI conditions and thus the plasma performance of the orally administered ibuprofen. While simulations can be performed and mechanistic insight gained from such simulations from current software, we need to further determine the dynamics of the GI variables controlling the dosage form transit, disintegration, dissolution absorption and metabolism along the GI tract in order to move from correlation to prediction (IVIVC → IVIVP). The obtained underlying cumulative dissolution profile derived from the GastroPlus^™^ simulator (Figure 13) and the simulated concentration–time profiles from the Phoenix WinNonlin^®^ platform (Figure 2) will serve as a reference to optimize our in-house biopredictive dissolution device, the Gastrointestinal Simulator (GIS). Establishing the link between biopredictive in vitro dissolution testing and mechanistic oral absorption modeling (i.e., physiologically-based biopharmaceutics modeling (PBBM)) opens an opportunity to potentially request biowaivers in the near future for orally administered drug products, regardless of its classification according to the BCS [47,48,49,50].

## Figures and Tables

**Table 1:** Physicochemical, biopharmaceutical and pharmacokinetic disposition properties to perform simulations in GastroPlus™ for ibuprofen.

Input Parameter	Value/Selection Dynamic Settings	Value/Selection Default Settings	Reference
**Physicochemical Properties**			
Molecular weight (g/mol)	206.29	206.29	ADMET Predictor 9.0
pKa (acidic)	4.54	4.54	ADMET Predictor 9.0
Octanol/water partition coefficient (log*P*)	3.65	3.65	ADMET Predictor 9.0
**Biopharmaceutics Properties**			
Human effective permeability (*P*_eff_) (× 10^−4^ cm/s)	4.1	4.1	[27]
Particle size radius (um)	62	62	In-house data
Dose volume (mL)	250	250	[13]
pH at reference solubility	6.2	6.2	[28]
Solubility at reference pH (mg/mL)	1.99	1.99	[28]
Solubility in Fasted state human gastric fluid (FaHGF) (mg/mL)—pH 1.46	0.0048	0.0048	[29]
Solubility in Fasted state human intestinal fluid (FaHIF) (duodenum) (mg/mL)—pH 3.74	0.0102	0.0102	[29]
Solubility in FaHIF (jejunum) (mg/mL)—pH 4.6	1.2	1.2	[30]
**Distribution and Clearance**			
Pharmacokinetic model	Two-compartmental	Two-compartmental	[31]
Clearance (L/h)	4.05	4.05	[31]
*K*_10_ (1/h)	1.16	1.16	[31]
*K*_12_ (1/h)	4.55	4.55	[31]
*K*_21_ (1/h)	3.46	3.46	[31]
**Advanced Compartmental and AbsorptionTransit model (ACAT^™^) Model Parameters**			
Gastric transit time (h)	2.04	0.25	[13]
Dynamic fluid volume model	Based on 100% of the volumes measured in human MRI study after drinking a glass of water (240 mL)	Default static values under the physiology tab ‘Human—Physiological—Fasted’	[26]
Dynamic pH model	Based on average pH values derived from gastric, duodenal and jejunal aspirated fluids after oral administration of 800 mg of ibuprofen	Default static values under the physiology tab ‘Human—Physiological—Fasted’	[13]

**Table 2:** Average absolute deviation percentage between predicted and experimental values of *C*_max_, *T*_max_ and *AUC* in the duodenum, jejunum and plasma.

Plasma	InVivo_GIS	InVivo_GISPlus
*C*_max_	48.3	19.8
*T*_max_	50.8	15.7
*AUC*	11.0	13.1
**Duodenum**	**InVivo_GIS**	**InVivo_GISPlus**
*C*_max_	50.8	15.7
*T*_max_	62.0	27.4
*AUC*	82.8	88.3
**Jejunum**	**InVivo_GIS**	**InVivo_GISPlus**
*C*_max_	46.91	25.47
*T*_max_	50.83	15.75
*AUC*	78.68	28.19

**Table 3:** InVivo_GISPlus individual and average parameter values. Median values and the fitted values for the average subjects are also shown for comparison. SD: standard deviation; CV: coefficient of variation. *K*_empt_ is the gastric emptying rate constant; K_TD and K_TJ represent the transit rate constants in the duodenal and jejunal compartment, respectively; K_Diss represent the intestinal dissolution rate constant; V1 and V2 represent the duodenal and jejunal residual volumes, respectively.

Parameter	*K*_EMPT_ (1/h)	K_TD (1/h)	K_TJ (1/h)	K_Diss mL/(ug*h)	V1 (mL)—Duodenal	V2 (mL)—Jejunal
**Average**	**0.84**	**1.99**	**0.13**	**0.10**	**155.11**	**70.64**
SD	1.27	3.01	0.25	0.36	128.44	80.48
CV%	151.84	150.83	196.68	361.28	82.81	113.93
B003V1	0.39	0.20	0.130	0.101	80.4	20.0
B004V1	0.08	0.59	0.059	1.48 × 10^−3^	73.9	18.5
B004V2	0.02	1.86	0.209	5.05 × 10^−3^	195.4	209.2
B005V1	0.51	0.22	0.010	1.61 × 10^−4^	15.3	2.5
B005V2	0.18	0.45	0.224	3.62 × 10^−4^	350.5	84.5
B006V1	0.95	1.84	0.042	3.51 × 10^−4^	123.2	64.6
B017V1	0.16	0.41	0.081	5.75 × 10^−3^	62.8	10.2
B017V2	0.07	0.08	0.036	1.11 × 10^−3^	77.6	19.5
B042V1	0.13	10.26	0.000	7.31 × 10^−2^	29.0	5.2
B042V2	0.05	0.19	0.086	1.52 × 10^−3^	150.0	80.6
B049V1	0.05	0.19	0.086	1.52 × 10^−3^	262.3	10.6
B049V2	0.05	0.19	0.086	1.52 × 10^−3^	150.0	80.6
B052V1	2.79	2.99	0.019	6.96 × 10^−4^	19.0	27.6
B053V1	1.24	1.79	0.039	3.48 × 10^−4^	137.8	215.8
B055V1	4.05	0.47	0.100	9.00 × 10^−5^	126.6	50.0
B055V2	3.65	0.07	0.001	4.33 × 10^−4^	119.6	93.4
B063V1	1.00 × 10^−3^	4.12	1.140	1.96 × 10^−3^	115.9	56.3
B065V1	0.20	2.51	0.092	0.121	500.0	283.1
B065V2	1.28	9.48	0.002	1.59	357.7	10.1
**Median**	**0.18**	**0.47**	**8.1 × 10^−2^**	**1.52 × 10^−3^**	**123.24**	**49.99**
Average Subject	0.45	0.47	7.9 × 10^−2^	9.34 × 10^−4^	136.53	56.89

**Table 4:** Default setting values for volume (mL) and pH in the GastroPlus™ simulator.

GI Compartment	Volume (mL)	pH	Transit Time (h)
1. Stomach	48.92	1.3	0.25
2. Duodenum	44.57	6	0.26
3. Jejunum 1	166.6	6.2	0.94
4. Jejunum 2	131	6.4	0.75
5. Ileum 1	102	6.6	0.58
6. Ileum 2	75.35	6.9	0.42
7. Ileum 3	53.57	7.4	0.29
8. Caecum	50.49	6.4	4.48
9. Ascending Colon	53.55	6.8	13.44

**Table 5:** Pharmacokinetic disposition parameters between observed and simulated data applying the default settings.

Pharmacokinetic (PK) Parameters	Observed Pharmacokinetic Data	Simulated Pharmacokinetic Data
Plasma *C*_max_ (µg/mL)	41.7	55.5
Plasma *T*_max_ (h)	3.00	1.31
Plasma *AUC*_0-∞_ (µg·h/mL)	259	189
Plasma *AUC*_0-28h_ (µg·h/mL)	255	189

**Table 6:** Pharmacokinetic disposition parameters between observed and simulated data applying the default settings.

Pharmacokinetic (PK) Parameters	Observed Pharmacokinetic Data	Simulated Pharmacokinetic Data
Plasma *C*_max_ (µg/mL)	41.7	32.5
Plasma *T*_max_ (h)	3.0	3.0
Plasma *AUC*_0-∞_ (µg·h/mL)	259	189
Plasma *AUC*_0-28h_ (µg·h/mL)	255	189

## Supplementary Materials

Both mathematical equations for simulations performed in Phoenix WinNonlin^®^ as well as the dynamic .cat files for simulations in GastroPlus^™^ are provided for the reader. These .cat files are compatible with all versions of GastroPlus^™^. All these files can be found at the following link for free: https://zenodo.org/record/3562430#.XfSmtOhKg-U.
